# Association of DOK3 and infiltrated tumor-associated macrophages with risk for the prognosis of *Porphyromonas gingivalis*-infected oral cancer: a 12-year data analysis of 200 patients from a tertiary teaching hospital, Urumqi, China

**DOI:** 10.1186/s12885-024-12300-y

**Published:** 2024-04-26

**Authors:** Chenxi Li, Muqiu Li, Wei Wei, Zhengye Wang, Jingwen Yu, Zhongcheng Gong

**Affiliations:** 1 Department of Oral and Maxillofacial Oncology & Surgery, School / Hospital of Stomatology, the First Affiliated Hospital of Xinjiang Medical University, No. 137 Liyushan South Road, Urumqi, 830054 P.R. China; 2 Stomatological Research Institute of Xinjiang Uygur Autonomous Region, Urumqi, 830054 China; 3 Hubei Province Key Laboratory of Oral and Maxillofacial Development and Regeneration, School of Stomatology, Tongji Medical College, Union Hospital, Huazhong University of Science and Technology, Wuhan, 430022 China; 4 Center for Disease Control and Prevention, Xinjiang Production and Construction Corps, Urumqi, 830092 China; 5 Department of Pathology, the First Affiliated Hospital of Xinjiang Medical University, Urumqi, 830054 China

**Keywords:** *Porphyromonasgingivalis*, Oral squamous cell carcinoma, Tumor microenvironment, Macrophages, Biochemistry, Survival analysis

## Abstract

**Background:**

While there is an understanding of the association between the expression of *Porphyromonas gingivalis* (*P. gingivalis*) and prognosis of oral squamous cell carcinoma (OSCC), significance specially to address the relevance between different immunohistochemical intensities of *P. gingivalis* and tumor-associated macrophages (TAMs) in OSCC tissue and related clinicopathologic characteristics has not been well investigated. The present study aimed to investigate the pathological features related to M2-TAM in *P. gingivalis*-infected OSCC and ascertain its clinical relevance with patients’ prognosis.

**Methods:**

A prospective cohort study was designed to comparatively analyze 200 patients from June 2008 to June 2020. Bioinformatics analyses were implemented to identify DOK3 as a key molecule and to appraise immunocyte infiltration using Gene Expression Omnibus and The Cancer Genome Atlas databases. Immunohistochemical evaluation was performed to analyze the association between the expression levels of *P. gingivalis*, DOK3, and M2-TAM and clinicopathological variables using Fisher’s exact test or Pearson’s chi-square test. Cox analysis was used to calculate hazard ratios (HR) with corresponding 95% confidence interval (CI) for various clinicopathological features. The Kaplan–Meier approach and log-rank test were used to plot the survival curves.

**Results:**

The expression level of *P. gingivalis* was positively associated with DOK3 and M2-TAMs expression level (*P* < 0.001). Parameters, including body mass index, clinical stage, recurrence, tumor differentiation, and *P. gingivalis*, DOK3, and M2-TAM immunoexpression levels, affected the prognosis of patients with OSCC (all *P* < 0.05). In addition, *P. gingivalis* (HR = 1.674, 95%CI 1.216–4.142, *P* = 0.012), DOK3 (HR = 1.881, 95%CI 1.433–3.457, *P* = 0.042), and M2-TAM (HR = 1.649, 95%CI 0.824–3.082, *P* = 0.034) were significantly associated with the 10-year cumulative survival rate.

**Conclusions:**

Elevated expression of *P. gingivalis* and DOK3 indicates M2-TAM infiltration and unfavorable prognosis of OSCC, and could be considered as three novel independent risk factors for predicting the prognosis of OSCC.

**Supplementary Information:**

The online version contains supplementary material available at 10.1186/s12885-024-12300-y.

## Introduction

Oral squamous cell carcinoma (OSCC), derived from epithelia, is the leading histopathological type, accounting for approximately up to 90% of all head and neck malignancies [1]. The anatomical distribution of OSCC includes the anterior two-thirds of the tongue, lower and upper gingiva, buccal mucosa, hard palate, floor of the mouth, retromolar triangle, and vermilion mucosa [2]. OSCC is characterized by its high local invasion and easy recurrence and metastasis, as well as relatively high morbidity and mortality (31,733 new cases and 15,745 deaths in 2022 in China, and 25,210 new cases and 4,452 deaths in 2022 in the USA) estimated through GLOBOCAN (http://globocan.iarc.fr/) [3]. In addition, owing to its poor prognosis, OSCC has gradually become a serious public health issue worldwide, despite the rapid development of multidisciplinary treatment [4]. Moreover, the 5-year overall survival rate for this disease has not significantly increased [4, 5] (Supplemental Fig. 1).

Since the recognition of a causal relationship between *Helicobacter pylori* infection and the occurrence of gastric cancer in the 1990s [6], a greater depth of understanding has been acquired with respect to bacterial carcinogenesis. However, the effect of the microbiome on oral cancer still remains unknown. As a precondition, many structures, including the nasal cavity, oral cavity, and sinuses (*e.g.*, frontal sinus, ethmoidal sinus, paranasal sinus, maxillary sinus and sphenoid sinus) in the oro-maxillofacial area constitute an ideal room, wherein the stable habitat of suitable salivary pH (6.5 to 7.5) and constant temperature (around 37℃) provided, whether for the growth of anaerobic bacteria or aerobes [7]. Some epidemiological studies have established chronic inflammatory diseases, such as periodontitis, as newly defined risk factors contributing to OSCC development [8–10]. *Porphyromonas gingivalis* (*P. gingivalis*), the most dominant bacteria in periodontal lesions, is a key pathogen that mediates the local immune inflammatory response in chronic periodontitis. More importantly, *P. gingivalis* tends to be positively associated with orodigestive cancers and its detection in patients with oral or esophageal SCC has adverse outcomes [11–14]. Additionally, *P. gingivalis* can adhere to gingival epithelial cells, interfere with the normal physiological metabolism of cells, and inhibit the cytotoxicity of programmed cell death [15]. Persistent exposure to *P. gingivalis* can also give rise to cell morphological changes, promote proliferative capacity with a higher S phase fraction in the cellular cycle, and facilitate cell invasion and migration [16].

It is a well-known fact that macrophage is the most plentiful and important tumor-infiltrating immune cell type, and it would be differentiated into M2-like tumor-associated macrophage (TAM) expressing CD68^+^, CD163^+^, and CD204^+^ in the local milieu of OSCC stromal spaces [17]. These TAMs have been analyzed in a broad spectrum of cancers with strong evidence demonstrating their carcinogenic function in the furtherance of metastasis and relapse. However, the potential interaction between *P. gingivalis* and TAMs and the effect of TAMs on the prognosis of *P. gingivalis*-infected OSCC remain unclear.

To the best of our knowledge, no study has evaluated the correlation between the immunoexpression and clinical significance of M2-TAMs in OSCC microenvironment of *P. gingivalis*-infection. Herein, we investigated this correlation through bioinformatics and biochemistry analyses, and downstream analysis of OSCC patient survival.

## Materials and methods

### Ethics

The study protocol was reviewed and approved by the Ethics Committee at the School/Hospital of Stomatology Xinjiang Medical University, Urumqi, PR China, with the onset of baselined data collection (approval no. IACUC20210706-11). The procedures in this study were completed in accordance with the standards set out in the Announcement of Helsinki and laboratory regulations of research in China. Written informed consent was obtained from all the patients.

### Patient selection

The current study included patients diagnosed with OSCC and their corresponding surgical specimens from June 2008 to June 2020 at the author’s affiliation. All included patients were treated using a multidisciplinary approach, and data were selected from the electronic medical records of the hospital information system. According to the most updated *American Joint Committee on Cancer/Union for International Cancer Control* (AJCC/UICC) guidelines (8th edition), the clinicopathological classification and staging of all recruited OSCC patients were assessed using the TNM system [*i.e.*, size of the primary tumor (T), involvement of locoregional lymph nodes (N), and distant metastases (M)], fully reflecting the extent of tumor growth in the whole body [18]. The inclusion criteria were as follows: *i*)*.* OSCC lesions are located in the tongue, gingiva, buccal mucosa, hard palate, floor of the mouth, retromolar triangle, and vermilion mucosa that are confirmed histopathologically; *ii*)*.* Patients who had not undergone any treatment previously; *iii*)*.* Cases of primary or recurrent tumors that received complete tumor resection with or without lymph node dissection; and *iv*)*.* At least three-year follow-up/survival materials were available.

**Fig. 1:**
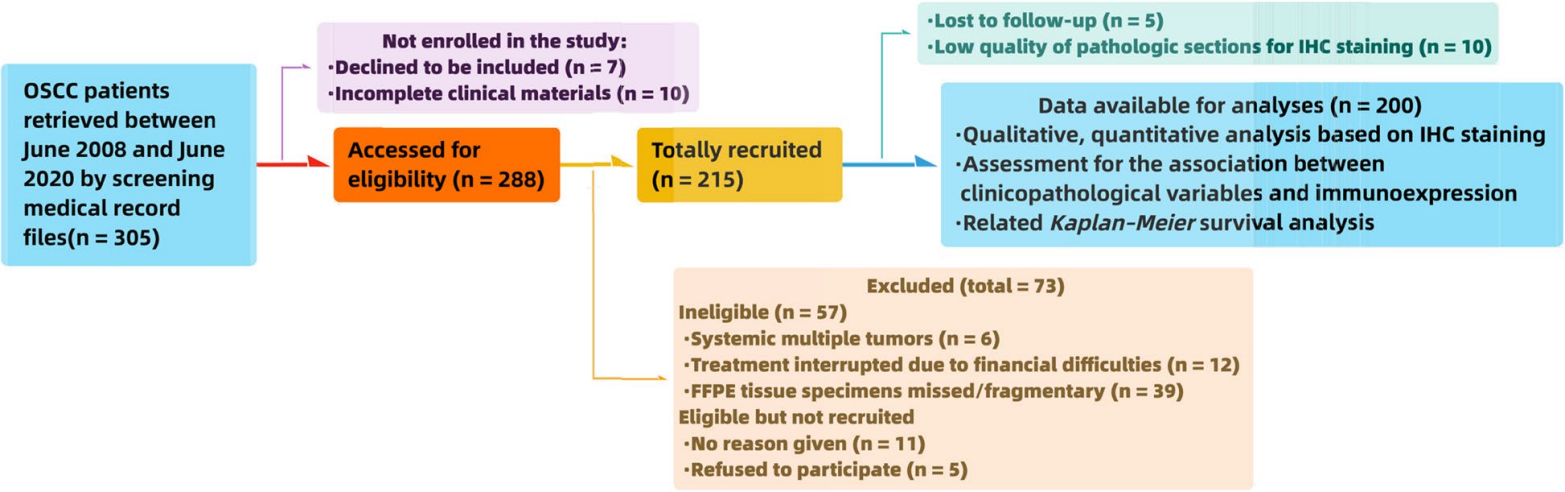
The flow diagram describing the subjects’ enrollment as well as the working plan

A total of 215 patients with OSCC met the inclusion criteria after carefully screening medical record files. All patients agreed to participate in the investigation; however, five patients were lost to follow-up, and the pathologic materials of ten potential participants were inadequate to perform immunohistochemistry (IHC). Finally, 200 patients with OSCC were enrolled in this clinicopathological correlation study. The study flowchart based on the STROBE (Strengthening the Reporting of Observational Studies in Epidemiology) statement [19] is shown in Fig. 1.

### Clinical data acquisition

The items collected included age, sex, alcohol consumption, tobacco smoking, diet, oral hygiene habits, behavioral swallowing, periodontal condition, anatomical distribution of the tumor, TNM staging of OSCC, tumor differentiation, recurrence, treatment regime (surgery with sequential chemotherapy and/or radiotherapy, postoperative adjuvant immunotherapy or radiotherapy or chemotherapy), and survival status.

### *P. gingivalis* assessment

*P. gingivalis* DNA was detected using PCR methods as established before [20]. To verify *P. gingivalis*-positive samples, one pair of 16S rDNA fragments were amplified from OSCC tissue and sequenced, confirmed by BLAST homology comparison (http://www.ncbi.nlm.nih.gov/BLAST) [12, 20].

### Bioinformatics analyses

To ascertain the appropriate target transcript, gene expression omnibus (GEO), a public functional genomics data repository (https://www.ncbi.nlm.nih.gov/geo/), was searched. Retrieval of terms was combined in the following search string to identify relevant array- and/or sequence-based data: “Homo sapiens” (organism) AND “Oral squamous cell carcinoma” OR “Macrophage” OR “*Porphyromonas gingivalis*” (study keyword) AND “Expression profiling by array” (experiment type). After a systematic review, gene expression sequencing datasets of GSE24897 [20] and GSE138206 [13] were collected for further analyses. Specifically, the GSE24897 dataset contained nine samples of *P. gingivalis*-infected macrophages (GSM612265-73) and three samples of uninfected macrophages (GSM612262-4); the GSE138206 dataset contained six samples of OSCC tissue (GSM4101925-30), six samples of tissue adjacent to cancer (GSM4101937-42), and six samples of contralateral normal tissue (GSM4101931-6). The probes were converted into corresponding gene symbols based on the annotation information in the platform.

The differentially expressed genes (DEGs) between the two screened datasets were analyzed using the Limma package in R software (version 4.2.2; R Foundation for Statistical Computing, Vienna, Austria). Probes with > 1 gene symbol or without corresponding gene symbols were considered as intersections or removed. For analyzing and heat-mapping DEGs, adjusted *P*-value (adj. *P*) < 0.01 and |log_2_FC (fold-change)|> 1 were considered to have statistically significant difference [13, 20]. Furthermore, to explore the pan-cancer landscape of macrophage infiltration, TIMER 2.0, an online tool (http://timer.cistrome.org/ or http://timer.comp-genomics.org/) was utilized to analyze the immune module [20].

### Histopathologic assessment

Tissues specimens were provided by Biobank of Oral Medicine and Pathology, Central Scientific and Research Institute of Stomatology, Xinjiang, China, and representative tissue specimens from 200 OSCC patients were obtained from archival formalin-fixed paraffin-embedded (FFPE) tumor blocks to construct tissue microarrays, as described in our previous work [13, 20]. OSCC tissue microarrays were consecutively cut into 4 μm sections and dried on IHC microscope slides (BC075, Biosharp, Beijing, China). The sections were deparaffinized using standard xylene and hydrated using a gradient of ethanol in water. Antigen repair was performed by heating the sections with EDTA antigenic retrieval buffer (pH 8.0). IHC staining of *P. gingivalis*, downstream of kinase 3 (DOK3), and M2-TAM was consistent as follows: anti-*P. gingivalis* monoclonal antibody (#ab225982, Abcam, Cambridge, UK) at 1:100 dilution [21]; anti-DOK3 monoclonal antibody (#ab236609, Abcam, Cambridge, UK) at 1:500 dilution [20]; and anti-M2-TAM monoclonal antibody (CD206^+^; #MA5-44,409, ThermoFisher Scientific, Waltham. MA, USA) at 1:200 dilution of incubation. The DAB chromogenic agent (#D5905, Sigma-Aldrich, Saint Louis. Missouri, USA) was used as the substrate for *P. gingivalis*, DOK3, and M2-TAM expression.

Each slice was independently assessed by two professional pathologists who were blinded to clinical data. The immunoreactivity of *P. gingivalis*, DOK3, and M2-TAM was measured according to a score that added the intensity of staining to the proportion of positive cells using ImageJ software (version 1.8.0; National Institutes of Health, Bethesda, Maryland, USA) [22].

### Statistical analysis

Data analysis was performed using R software (version 4.2.2; R Foundation for Statistical Computing, Vienna, Austria). Clinicopathologic characteristics of included patients were described as absolute frequency (percentage), and bivariate analysis to evaluate the association between clinicopathologic variables and *P. gingivalis*, DOK3, and M2-TAM immunoexpression levels in the tumor microenvironment (TME) of OSCC was determined using the Chi-square or Fisher’s exact test. The correlation between the levels of *P. gingivalis*, DOK3, and M2-TAM in specimens from patients with OSCC by immunohistochemical staining assays was analyzed using Pearson’s correlation. The Kaplan–Meier method was used to estimate the cumulative survival rate (CSR) probability over 10 years, and the log-rank test was used to compare prognosis among patients. Univariate and multivariate Cox proportional hazards regression models were employed to calculate the relevant hazard ratios (HR) with their 95% confidence intervals (CI). All tests were two-sided and *P* values less than 0.05 were considered statistically significant.

## Results

### General information of study population

In total, 200 samples form patients newly diagnosed with OSCC (49–81 years; mean age, 63.29 ± 6.42 years) were prospectively analyzed. To eliminate the possible interference of sex factors, we achieved a 1:1 sex ratio (100 males and 100 females). Most patients with OSCC required extensive treatment, including radiotherapy, chemoradiotherapy and/or surgical resection. Patients with advanced or metastatic OSCC were treated with palliative chemoradiotherapy. With respect to living status, most patients (66.5%) were alive at the time of the present prospective analysis. Data concerning on M stage are not shown because no patients had distant metastases at the time of physical examination. Clinicopathologic features of the patients are listed in Supplemental Table 1.

### Identification of DOK3 as a key DEG in the TME of OSCC infected with *P. gingivalis*

**Fig. 2:**
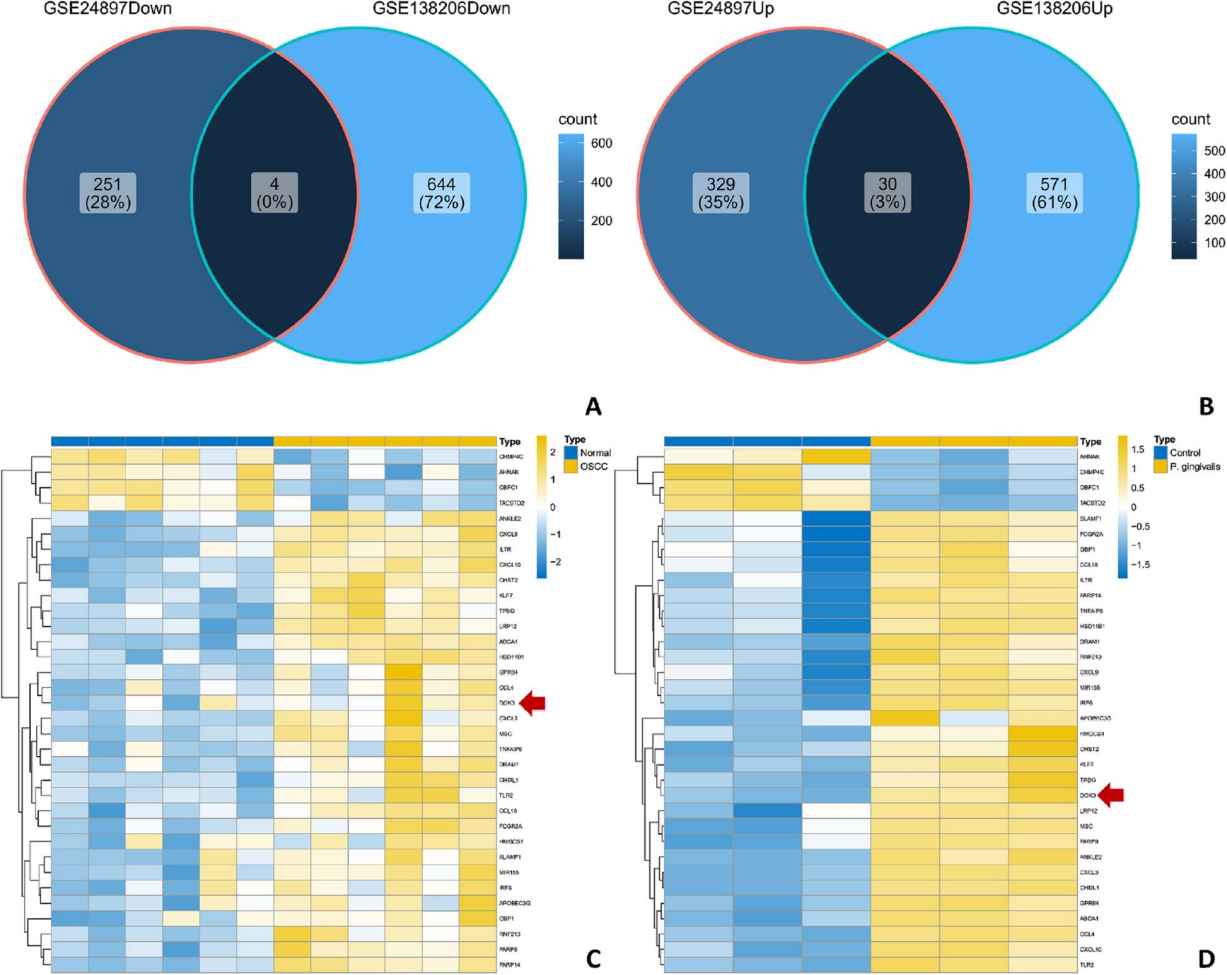
Venn plots and heat maps of DEGs. DEG with |log_2_FC|> 1 and adj. *P* < 0.01 were selected in the expression profile of GSE138206 and GSE24897. DOK3 was one of the co-DEGs included in the two collections. **A** 4 genes in common among all downregulated genes. **B** 30 genes in common among all upregulated genes. **C** Hierarchical clustering of 34 common genes in GSE138206. **D** Hierarchical clustering of 34 common genes in GSE24897

**Fig. 3:**
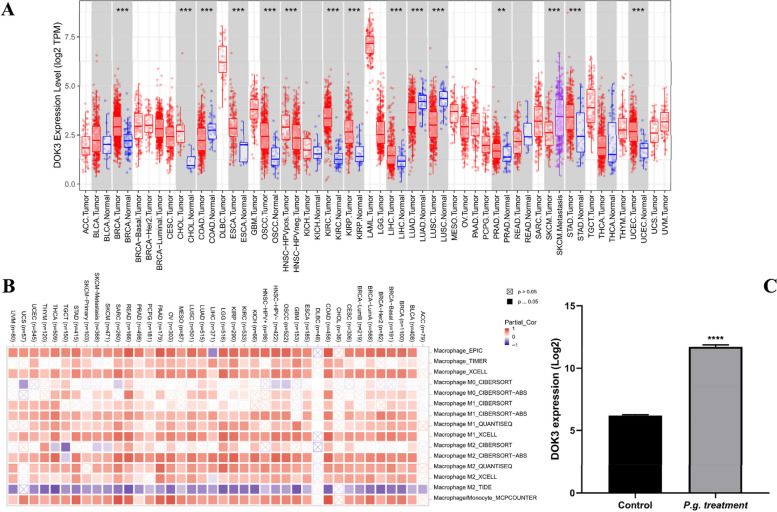
The relationship between DOK3 expression and cancer microenvironment. **A** The differential expression map of DOK3 on pan-cancer data showing a significant increase in OSCC than that in normal tissue. **B** Correlation between DOK3 and infiltrating tumor-associated macrophage. **C** DOK3 expression in *P. gingivalis* infection of macrophages microarray. Statistical differences were considered significant if * P < 0.05; ** P < 0.01; *** P < 0.001; **** P < 0.0001

A total of 1,863 DEGs (903 genes down and 960 genes up) were identified after standardizing the expression profile of sequencing data, and the overlap of the two datasets (GSE138206 and GSE24897) collecting 30 upregulated and 4 downregulated common DEGs is illustrated in Fig. 2A and B and Supplemental Table 2. DOK3 was one of the hub genes included in the two datasets based on hierarchical clustering (Fig. 2C, D). To further demonstrate the performance of DOK3 in TME, pan-cancer analysis of macrophage infiltration analysis was performed using The Cancer Genome Atlas (TCGA) database (https://portal.gdc.cancer.gov/). The analyses revealed that the expression of a single gene (DOK3) was significantly increased in various cancers, including OSCC (*P* < 0.001) (Fig. 3A), and DOK3 expression could be positively correlated with the M2-TAMs infiltration in OSCC (*r* = 0.72, *P* < 0.001) (Fig. 3B), and the effect of DOK3 expression on M2-TAMs infiltration was significantly increased after *P. gingivalis* treatment (*P* < 0.0001) (Fig. 3C).

### IHC analysis of *P. gingivalis* in OSCC patients and associations with clinicopathological parameters

**Fig. 4:**
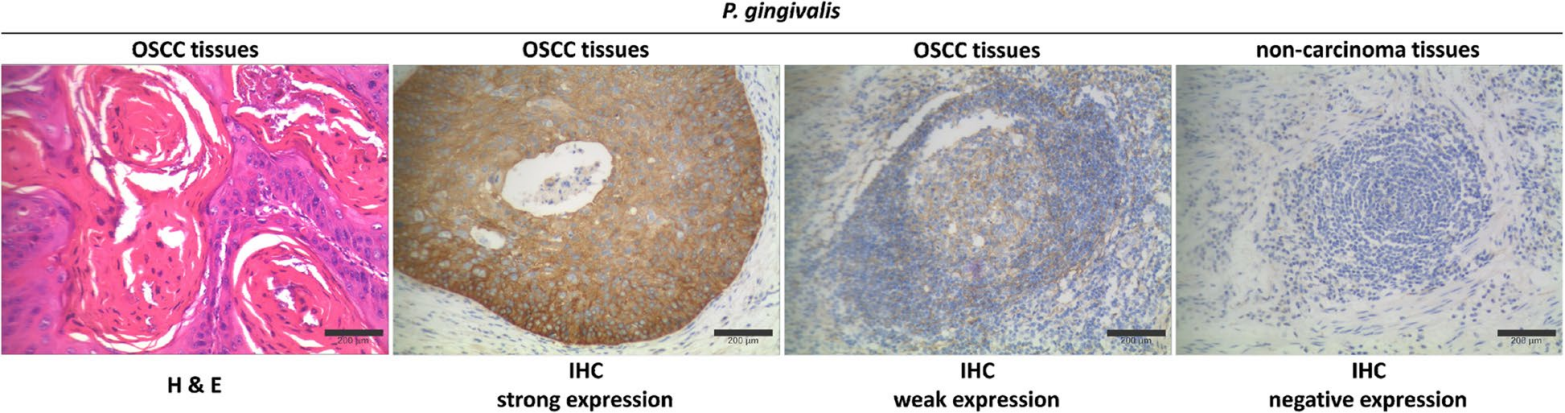
H & E staining of *P. gingivalis* in OSCC tissues; strong immunoexpression of *P. gingivalis* in OSCC tissues; weak immunoexpression of *P. gingivalis* in OSCC tissues; and negative *P. gingivalis* expression in adjacent non-carcinoma tissues. (original magnification × 200)

*P. gingivalis* was detected in all 200 (100%) OSCC specimens, with predominant immunostaining in the cytoplasm of neoplastic cells. Of these, 139 cases (69.5%) were strongly positive and 61 cases (30.5%) were weakly positive, whereas matched adjacent normal tissues were negative (Fig. 4). The associations between *P. gingivalis* expression and clinicopathological characteristics are summarized in Table 1. Strong immunoexpression of *P. gingivalis* was significantly associated with tobacco smoking, poor oral hygiene habits, poor periodontal condition, larger tumor size (diameter ≥ 3 cm), poor tumor differentiation, advanced T stage and clinical stage, neck lymph node metastasis, and death (all *P* < 0.05). No other significant associations were observed with the remaining variables (Table 1).

**Table 1:** Immunohistochemical expression of *P. gingivalis* in 200 patients with oral squamous cell carcinoma according to the clinical data and follow-up

Variable	*P. gingivalis*		*P* value
	**Weak (%)**	**Strong (%)**	
**Sex**			0.282
Male	27 (44.3)	73 (52.5)	
Female	34 (55.7)	66 (47.5)	
**Age (yr)**			0.580
< 60	24 (39.3)	49 (35.3)	
≥ 60	37 (60.7)	90 (64.7)	
**Survival status**			0.002^**^
Alive	50 (82.0)	83 (59.7)	
Dead	11 (18.0)	56 (40.3)	
**Tobacco smoking**			0.000^***^
No	31 (50.8)	37 (26.6)	
Yes	30 (49.2)	102 (73.4)	
**Alcohol consumption**			0.160
No	32 (52.5)	58 (41.7)	
Yes	29 (47.5)	81 (58.3)	
**Baseline severe dysphagia (Gr.3–6)**^a^			0.905
No	50 (82.0)	79 (56.8)	
Yes	11 (18.0)	60 (43.2)	
**Diet**			0.181
Vegetarian	12 (19.7)	7 (5.0)	
Non-vegetarian	49 (80.3)	132 (95.0)	
**Milk & dairy products**			0.026
Never	0	2 (1.4)	
Less than once a week	7 (11.5)	22 (15.8)	
More than once a week	54 (88.5)	115 (82.8)	
**T stage**^b^			0.000^***^
T1 ~ T2	47 (77.0)	72 (51.8)	
T3 ~ T4	14 (23.0)	67 (48.2)	
**N stage**^b^			0.025^*^
N0	48 (78.7)	87 (62.6)	
N ( +)	13 (21.3)	52 (37.4)	
**Clinical stage**^b^			0.000^***^
I ~ II	44 (72.1)	56 (40.3)	
III ~ IV	17 (27.9)	83 (59.7)	
**Recurrence**			0.298
Yes	7 (11.5)	24 (17.3)	
No	54 (88.5)	115 (82.7)	
**Tumor size (cm)**			0.014^*^
< 3	36 (59.0)	55 (39.6)	
≥ 3	25 (41.0)	84 (60.4)	
**Differentiation**			0.003^**^
Well	49 (80.33)	80 (57.6)	
Moderate	11 (18.03)	38 (27.3)	
Poor	1 (1.64)	21 (15.1)	
**Periodontal condition**			0.000^***^
Well	28 (45.9)	28 (20.1)	
Poor	33 (54.1)	111 (79.9)	
**Oral hygiene habits**^c^			0.019^*^
Good	16 (26.2)	51 (36.7)	
Average	41 (67.2)	69 (49.6)	
Bad	4 (6.6)	19 (13.7)	
**Treatment**			0.351
Surgery	16 (26.2)	47 (33.8)	
Radiotherapy	22 (36.1)	37 (26.6)	
Chemoradiation/comprehensive	23 (37.7)	55 (39.6)	

*Abbreviations*: *cm* centimeter, *Gr* Grade, *yr* year

Statistically significant (^*^P < 0.05, ^**^P < 0.01, ^***^P < 0.001)

^a^Grade of dysphagia according to the standard symptom scale (10.4103/0973–1482.63563). In subjective dysphagia, evaluation score ranges from 0 to 6. Score 0 suggests no dysphagia and score 6 suggests ‘nothing by mouth’

^b^According to the 8th edition of the American Joint Committee on Cancer/ the International Union Against Cancer staging system

^c^The composite oral hygiene score [23], ranging from 0 to 6 (with a score of 4 or more indicating poor oral hygiene; 2 to 3, reasonable; 1 or less indicating good hygiene), aimed to capture oral hygiene habits and intra-oral examination findings for each study participant by summing up the following states: bleeding gums (no = 0, yes = 1); frequency of cleaning teeth (> 2 times a day = 0, ≤ once a day = 1); instrument used for cleaning (toothbrush = 0, finger or other = 1); substance used for cleaning (toothpaste/toothpowder = 0, other = 1); wearing dentures (no = 0, yes = 1); dental check-ups (rare = 0, only when in pain = 1); missing teeth (≤ 5 = 0, > 5 = 1)

### IHC analysis of DOK3 and M2-TAMs in *P. gingivalis*-infected TME of OSCC patients

**Fig. 5:**
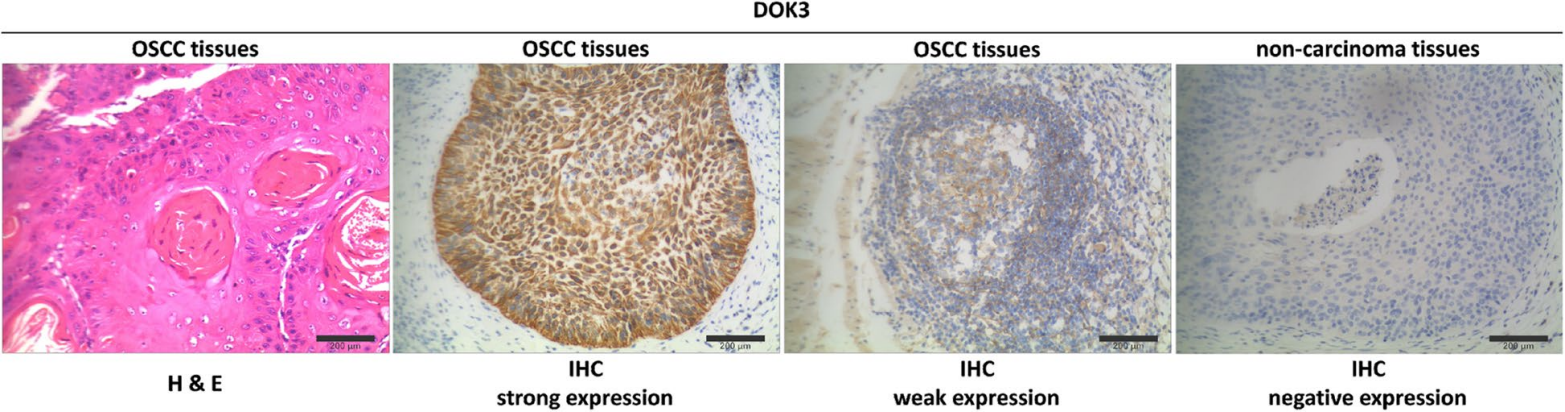
H & E staining of DOK3 in OSCC tissues^†^; strong immunoexpression of DOK3 in OSCC tissues; weak immunoexpression of DOK3 in OSCC tissues; and negative DOK3 expression in adjacent non-carcinoma tissues. (original magnification × 200). ^†^These OSCC tissues were confirmed as strong expression of *P. gingivalis* before

**Fig. 6:**
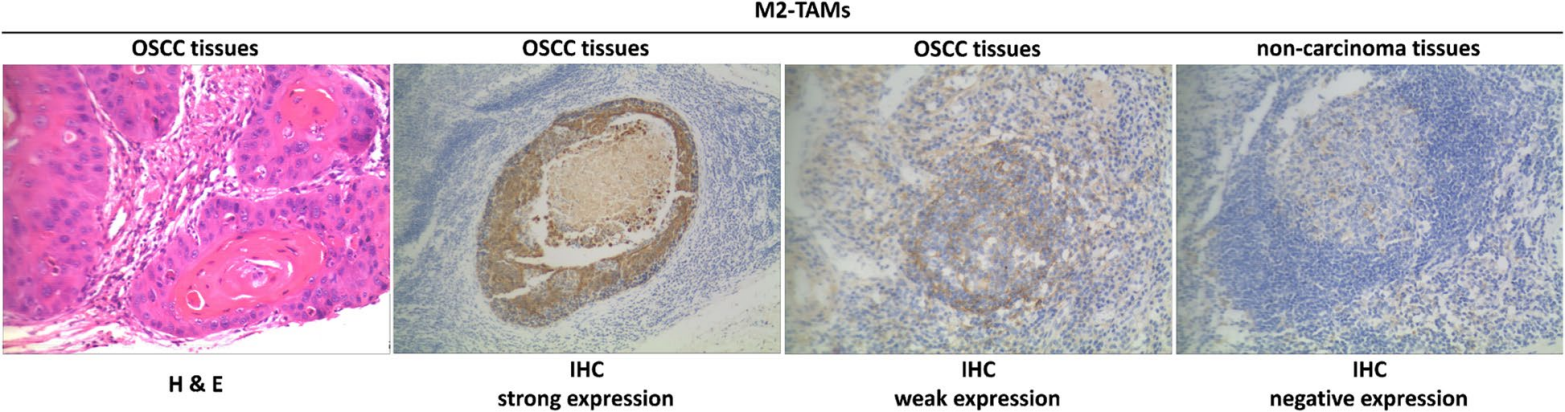
H & E staining of M2-TAMs in OSCC tissues^†^; strong immunoexpression of M2-TAMs in OSCC tissues; weak immunoexpression of M2-TAMs in OSCC tissues; and negative M2-TAMs expression in adjacent non-carcinoma tissues. (original magnification × 200). ^†^These OSCC tissues were confirmed as strong expression of *P. gingivalis* before

A total of 139 OSCC samples confirmed with high expression level of *P. gingivalis* were selected for further IHC examination of DOK3 and M2-TAMs, respectively. Of these 139 samples, 92 cases (66.2%) had strongly positive expression of DOK3 and 78 cases (56.1%) had strongly positive expression of CD206^+^ TAMs (M2-type) (Figs. 5 and 6), while 47 cases (33.8%) had weakly positive expression of DOK3 and 61 cases (43.9%) had weakly positive expression of CD206^+^ TAMs respectively (Figs. 5 and 6).

Several variables, including old age (≥ 60 year), tobacco smoking, poor periodontal condition, poor oral hygiene habits, severe dysphagia, larger tumor size (diameter ≥ 3 cm), advanced T stage, and death exhibited significant relationships with strong DOK3 expression (all *P* < 0.05) (Table 2). Moreover, strong staining of CD206^+^ TAMs was significantly associated with female sex, alcohol consumption, poor oral hygiene habits, severe dysphagia, advanced T stage and clinical stage, and chemoradiotherapy/comprehensive treatment (all *P* < 0.05) (Table 2).

**Table 2:** Immunohistochemical expression of DOK3 and M2-TAMs in 139 OSCC patients with strong *P. gingivalis* staining according to the clinical data and follow-up

Variable	DOK3		*P* value	M2-TAMs		*P* value
	**Weak (%)**	**Strong (%)**		**Weak (%)**	**Strong (%)**	
**Sex**			0.406			0.085^*^
Male	27 (57.4)	46 (50.0)		27 (44.3)	46 (59.0)	
Female	20 (42.6)	46 (50.0)		34 (55.7)	32 (41.0)	
**Age (yr)**			0.042^*^			0.372
< 60	22 (46.8)	27 (29.3)		24 (39.3)	25 (32.1)	
≥ 60	25 (53.2)	65 (70.7)		37 (60.7)	53 (67.9)	
**Survival status**			0.004^**^			0.052
Alive	36 (76.6)	47 (51.1)		42 (68.9)	41 (52.6)	
Dead	11 (23.4)	45 (48.9)		19 (31.1)	37 (47.4)	
**Tobacco smoking**			0.026^*^			0.632
No	18 (38.3)	19 (20.7)		15 (24.6)	22 (28.2)	
Yes	29 (61.7)	73 (79.3)		46 (75.4)	56 (71.8)	
**Alcohol consumption**			0.614			0.000^***^
No	21 (44.7)	37 (40.2)		35 (57.4)	23 (29.5)	
Yes	26 (55.3)	55 (59.8)		26 (42.6)	55 (70.5)	
**Baseline severe dysphagia (Gr.3–6)**			0.000^***^			0.028^*^
No	38 (80.9)	41 (44.6)		43 (70.5)	36 (46.2)	
Yes	9 (19.1)	51 (55.4)		18 (29.5)	42 (53.8)	
**Diet**			0.138			0.097
Vegetarian	7 (14.9)	0		5 (8.2)	2 (2.6)	
Non-vegetarian	40 (85.1)	92 (100)		56 (91.8)	76 (97.4)	
**Milk & dairy products**			0.677			0.240
Never	1 (2.1)	1 (1.1)		0	2 (2.6)	
Less than once a week	10 (21.3)	12 (13.0)		10 (16.4)	12 (15.4)	
More than once a week	36 (76.6)	79 (85.9)		51 (83.6)	64 (82.0)	
**T stage**			0.023^*^			0.017^*^
T1 ~ T2	31 (66.0)	42 (45.7)		39 (63.9)	34 (43.6)	
T3 ~ T4	16 (34.0)	50 (54.3)		22 (36.1)	44 (56.4)	
**N stage**			0.088			0.319
N0	34 (72.3)	53 (57.6)		41 (67.2)	46 (59.0)	
N ( +)	13 (27.7)	39 (42.4)		20 (32.8)	32 (41.0)	
**Clinical stage**			0.697			0.025^*^
I ~ II	20 (42.6)	36 (39.1)		31 (50.8)	25 (32.1)	
III ~ IV	27 (57.4)	56 (60.9)		30 (49.2)	53 (67.9)	
**Recurrence**			0.675			0.114
Yes	9 (19.1)	15 (16.3)		7 (11.5)	17 (21.8)	
No	38 (80.9)	77 (83.7)		54 (88.5)	61 (78.2)	
**Tumor size (cm)**			0.000^***^			0.619
< 3	29 (61.7)	27 (29.3)		26 (42.6)	30 (38.5)	
≥ 3	18 (38.3)	65 (70.7)		35 (57.4)	48 (61.5)	
**Differentiation**			0.256			0.083
Well	33 (70.2)	47 (51.1)		42 (68.85)	38 (48.7)	
Moderate	10 (21.3)	28 (30.4)		14 (22.95)	24 (30.8)	
Poor	4 (8.5)	17 (18.5)		5 (8.20)	16 (20.5)	
**Oral hygiene habits**			0.020^*^			0.044^*^
Good	5 (10.6)	15 (16.3)		8 (13.1)	12 (15.4)	
Average	15 (31.9)	36 (39.1)		22 (36.1)	29 (37.2)	
Bad	27 (57.5)	41 (44.6)		31 (50.8)	37 (47.4)	
**Periodontal condition**			0.013^*^			0.248
Well	15 (31.9)	13 (14.1)		15 (24.6)	13 (16.7)	
Poor	32 (68.1)	79 (85.9)		46 (75.4)	65 (83.3)	
**Treatment**			0.175			0.035^*^
Surgery	11 (23.4)	36 (39.1)		19 (31.1)	28 (35.9)	
Radiotherapy	14 (29.8)	23 (25.0)		11 (18.0)	26 (33.3)	
Chemoradiation/comprehensive	22 (46.8)	33 (35.9)		31 (50.8)	24 (30.8)	

*Abbreviations*: *cm* centimeter, *Gr* Grade, *OSCC* oral squamous cell carcinoma, *TAM* tumor-associated macrophage, *yr* year

Statistically significant (*P < 0.05, **P < 0.01, ***P < 0.001)

Notably, the expression level of *P. gingivalis* positively correlated with DOK3 expression level and M2-TAMs expression level, as determined by Pearson’s correlation analysis (all *P* < 0.001) (Table 3).

**Table 3:** Correlations between the immunohistochemical expression of *P. gingivalis* with DOK3 and M2-TAMs respectively

Variable	DOK3		*P* value	M2-TAMs		*P* value
	**Weak (%)**	**Strong (%)**		**Weak (%)**	**Strong (%)**	
***P. gingivalis***			< 0.001			< 0.001
Weak	38 (62.3)	23 (37.7)		41 (71.9)	16 (28.1)	
Strong	47 (33.8)	92 (66.2)		61 (42.7)	82 (57.3)	
Total	85 (42.5)	115 (57.5)		102 (51.0)	98 (49.0)	

*Abbreviations*: *P. gingivalis Porphyromonas gingivalis*, *TAM* tumor-associated macrophage

### Impact of *P. gingivalis*, DOK3, and M2-TAM immunoexpression on cumulative survival estimates of OSCC patients

The results of the Cox univariate proportional hazards regression showed that age, periodontal condition, body mass index (BMI), clinical stage, T stage, recurrence, tumor size, tumor differentiation, neck dissection, poor oral hygiene habits, *P. gingivalis*, DOK3, and M2-TAM immunoexpression levels were significantly different (all *P* < 0.05) (Table 4). Consequently, these variables were chosen as covariants in the multivariate Cox analysis.

**Table 4:** Univariate analysis of the Cox proportional-hazards regression model for patients with oral squamous cell carcinoma

Variable		Total no. = 200	Survival rate (%)	HR value	95% CI	*P* value
**Sex**	Male	100	50.0	Ref	Ref	Ref
	Female	100	50.0	0.824	0.436–1.618	0.847
**Age (yr)**	≥ 60	127	63.5	Ref	Ref	Ref
	< 60	73	36.5	0.662	0.296–0.963	0.028*
**Survival status**	Alive	133	66.5	Ref	Ref	Ref
	Dead	67	33.5	0.982	0.216–2.052	0.729
**Tobacco smoking**	No	68	34.0	Ref	Ref	Ref
	Yes	132	66.0	1.046	0.612–1.894	0.853
**Alcohol consumption**	No	90	45.0	Ref	Ref	Ref
	Yes	110	55.0	0.516	0.213–1.021	0.092
**Diet**	Vegetarian	19	9.5	Ref	Ref	Ref
	Non-vegetarian	181	90.5	0.626	0.240–1.117	1.701
**Milk & dairy products**	Never	2	1.0	Ref	Ref	Ref
	Less than once a week	29	14.5	0.552	0.295–0.997	1.692
	More than once a week	169	84.5	0.755	0.262–1.229	0.183
**BMI**	< 22.5	132	66.0	Ref	Ref	Ref
	≥ 22.5	68	34.0	0.646	0.313–0.947	0.032^*^
**Baseline severe dysphagia (Gr.3–6)**	No	129	64.5	Ref	Ref	Ref
	Yes	71	35.5	1.579	1.002–1.859	0.677
**T stage**	T1 ~ T2	119	59.5	Ref	Ref	Ref
	T3 ~ T4	81	40.5	1.441	0.522–1.619	0.002^**^
**N stage**	N0	135	67.5	Ref	Ref	Ref
	N ( +)	65	32.5	0.968	0.248–1.635	0.818
**Clinical stage**	I ~ II	100	50.0	Ref	Ref	Ref
	III ~ IV	100	50.0	1.395	0.573–1.726	0.016^*^
**Recurrence**	Yes	31	15.5	Ref	Ref	Ref
	No	169	84.5	0.813	0.546–1.024	0.005^**^
**Tumor size (cm)**	< 3	91	45.5	Ref	Ref	Ref
	≥ 3	109	54.5	0.822	0.072–1.098	0.022^*^
**Differentiation**	Well	129	64.5	Ref	Ref	Ref
	Moderate	49	24.5	1.025	0.717–1.446	0.000^***^
	Poor	22	11.0	1.806	0.428–2.352	0.017^*^
**Neck dissection**	No	137	68.5	Ref	Ref	Ref
	Yes	63	31.5	0.923	0.625–2.163	0.027^*^
**Oral hygiene habits**	Good	67	33.5	Ref	Ref	Ref
	Average	110	55.0	0.995	0.026–1.655	0.066
	Bad	23	11.5	1.229	0.183–1.975	0.018^*^
**Periodontal condition**	Well	56	28.0	Ref	Ref	Ref
	Poor	144	72.0	1.437	0.846–1.895	0.043^*^
***P. gingivalis***	Weak	61	30.5	Ref	Ref	Ref
	Strong	139	69.5	1.137	0.135–1.503	0.026^*^
**DOK3**^**a**^	Weak	47	33.8	Ref	Ref	Ref
	Strong	92	66.2	1.085	0.156–1.514	0.018^*^
**M2-TAM**^**b**^	Weak	61	43.9	Ref	Ref	Ref
	Strong	78	56.1	1.419	0.283–1.862	0.013^*^

*Abbreviations*: *BMI* body mass index, *CI* confidence interval, *cm* centimeter, *Gr* Grade, *HR* hazard ratios, *P. gingivalis Porphyromonas gingivalis*, *Ref* reference, *TAM* tumor-associated macrophage, *yr* year

Statistically significant (^*^P < 0.05, ^**^P < 0.01, ^***^P < 0.001)

^a^The sample size for DOK3 detection was 139 OSCC patients with strong immunostaining for *P. gingivalis* (Table 2)

^b^The sample size for M2-TAM detection was 139 OSCC patients with strong immunostaining for *P. gingivalis* (Table 2)

**Fig. 7:**
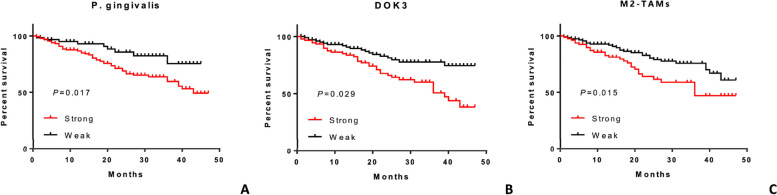
Kaplan–Meier curves for the overall survival in patients with oral squamous cell carcinoma. **A** Survival probability between strong and weak immunoexpression of *P. gingivalis*. **B** Survival probability between strong and weak immunoexpression of DOK3. **C** Survival probability between strong and weak immunoexpression of M2-TAMs

Cox multivariate analysis indicated that clinicopathological parameters including BMI, clinical stage, recurrence, tumor differentiation, and *P. gingivalis*, DOK3, and M2-TAM immunoexpression levels affected the prognosis of patients with OSCC (all *P* < 0.05) (Table 5). Importantly, the immunoexpression levels of *P. gingivalis* (HR = 1.674, 95%CI 1.216–4.142, *P* = 0.012), DOK3 (HR = 1.881, 95%CI 1.433–3.457, *P* = 0.042), and M2-TAM (HR = 1.649, 95%CI 0.824–3.082, *P* = 0.034), they were novel independent risk factors for the prognosis of patients with OSCC, which were significantly associated with the 10-year cumulative survival rate (Table 5 and Fig. 7).

**Table 5:** Multivariate analysis of the Cox proportional-hazards regression model for 200 patients with oral squamous cell carcinoma

Variable		*β* coef	S.E	Wald	HR (95% CI)	*P* value
**Age (yr)**	≥ 60	Ref	Ref	Ref	Ref	Ref
	< 60	0.053	0.031	3.162	0.723 (0.311–1.261)	0.283
**BMI**	< 22.5	Ref	Ref	Ref	Ref	Ref
	≥ 22.5	-0.653	0.271	5.358	0.554 (0.302–0.985)	0.034^*^
**T stage**	T1 ~ T2	Ref	Ref	Ref	Ref	Ref
	T3 ~ T4	0.061	0.041	3.862	1.524 (0.471–2.128)	0.052
**Clinical stage**	I ~ II	Ref	Ref	Ref	Ref	Ref
	III ~ IV	0.264	0.209	6.764	1.628 (0.831–2.315)	0.021^*^
**Recurrence**	Yes	Ref	Ref	Ref	Ref	Ref
	No	0.864	0.294	12.683	0.897 (0.629–1.246)	0.003^**^
**Tumor size (cm)**	< 3	Ref	Ref	Ref	Ref	Ref
	≥ 3	0.648	0.273	2.081	0.914 (0.127–1.874)	0.648
**Differentiation**	Well	Ref	Ref	Ref	Ref	Ref
	Moderate	0.527	0.341	13.057	1.016 (0.702–1.336)	0.000^***^
	Poor	0.954	0.527	3.152	1.811 (0.849–2.717)	0.381
**Neck dissection**	No	Ref	Ref	Ref	Ref	Ref
	Yes	0.421	0.137	4.689	1.624 (0.348–2.526)	0.757
**Periodontal condition**	Well	Ref	Ref	Ref	Ref	Ref
	Poor	0.682	0.426	1.025	1.841 (1.569–3.437)	0.946
**Oral hygiene habits**	Good	Ref	Ref	Ref	Ref	Ref
	Average	0.417	0.603	3.152	1.006 (0.477–1.895)	0.058
	Bad	0.530	0.916	5.158	1.915 (1.002–2.216)	0.055
***P. gingivalis***	Weak	Ref	Ref	Ref	Ref	Ref
	Strong	0.746	0.319	6.319	1.674 (1.216–4.142)	0.012^*^
**DOK3**	Weak	Ref	Ref	Ref	Ref	Ref
	Strong	0.547	0.307	5.527	1.881 (1.433–3.457)	0.042^*^
**M2-TAM**	Weak	Ref	Ref	Ref	Ref	Ref
	Strong	0.316	0.208	5.942	1.649 (0.824–3.082)	0.034^*^

Statistically significant (^*^P < 0.05, ^**^P < 0.01, ^***^P < 0.001)

*Abbreviations*: *BMI* body mass index, *CI* confidence interval, *cm* centimeter, *coef* coefficient, *HR* hazard ratios, *P. gingivalis Porphyromonas gingivalis*, *Ref* reference, *S.E* standard error, *TAM* tumor-associated macrophage, *yr* year

## Discussion

OSCC is the most common type of oral malignancy, which usually originates from precancerous lesions of the oral mucosa, and metastasis accounts for its poor prognosis. Although multidisciplinary and comprehensive treatment strategies have brought substantial progresses in prognostic outcomes, OSCC has a profound influence on human health and quality of life owing to its high morbidity and mortality [24]. A large amount of evidence has indicated that alcohol, tobacco products, areca nut, betel quid chewing, and genetic alterations are causative factors implicated in OSCC progression [25]. The microbiome has entered the perspective of the academic community since the commencement of the new millennium [7], and its role in the promotion of OSCC has gradually become a novel area of research. Although a definitive link between oral microflora and OSCC is yet to be established, accumulating evidence demonstrates that a variety of microbiological agents can also contribute to the progression of oral carcinogenesis in the presence of definitive risk factors such as alcoholism and smoking [7, 26]. To our knowledge, this is the first qualitative study adopting a comprehensive, theory-driven approach to investigate the the association between different expression levels of *P. gingivalis* and infiltrated M2-TAMs and the clinical significance impacting on the prognosis of patients with OSCC in Urumqi, China.

*P. gingivalis* is an important opportunistic pathogenic bacterium that can exist, survive and reproduce in the cytoplasm of infected cells [27]. In this present study, *P. gingivalis* immunostaining was observed in all OSCC specimens (including 61 weakly expressed and 139 strongly expressed) but was almost absent in matched adjacent non-carcinoma tissues. The vast majority of previous clinical studies have revealed that *P. gingivalis* is a high-risk factor for OSCC linked to a worse outcome by measuring OSCC subjects versus healthy individuals [12, 13, 28]. However, few studies have attempted to quantify different strengths, and show differences in clinical outcomes by discriminating between strong and weak immunoexpression levels. While our investigation, followed by our recent findings [13], found that strong immunoexpression of *P. gingivalis* was significantly associated with the presence of tobacco smoking, poor oral hygiene habits, poor periodontal condition, larger tumor size (diameter ≥ 3 cm), poor tumor differentiation, advanced T stage and clinical stage, neck lymph node metastasis, and death (all *P* < 0.05). Overall, a few studies have also reported that the detection of *P. gingivalis* in both serum and saliva is correlated with a high risk of oral cancer [12, 14, 29]. Another prospective case–control study also showed that overall oral microbiome composition was associated with risk of SCC of the head and neck, and greater abundance of periodontitis-associated bacteria was more likely to be present in the cancer cases through bacterial 16S rRNA gene sequencing [30]. These conclusions should be interpreted with caution because *P. gingivalis* is an opportunistic periodontopathic bacterium that can also live in the oral cavity, salivary glands, and peripheral blood circulation of healthy volunteers [31, 32]. Hence, the provenance of *P. gingivalis* in OSCC tissues and its abundance may explain the problem.

The products of *P. gingivalis* and/or its metabolic by-products significantly contribute to the development of oral carcinogenesis via their involvement in chronic inflammation. The OSCC microenvironment often resembles that of chronic inflammation induced by the dynamic interplay between tumor cells and the milieu it belongs to [33]. In addition, this proinflammatory microenvironment can increase the number of CD66b^+^ neutrophils discovered in OSCC, and these neutrophils are positively associated with poor prognosis, as reported in our previous investigation [13]. TAM constitutes the largest number of immune cells, accounting for up to 50% of solid neoplasms. Additionally, TAM, often referred to as M2-like macrophages, are capable of promoting tumor angiogenesis, immunosuppression, and metastasis in cancer progression [34]. Nevertheless the available scientific literature regarding the topic on “pathological features of TAMs related to tumor immunity and its clinical significance of *P. gingivalis*-infected OSCC” is nonexistent. To reasonably recognize a crucial indicator, we performed bioinformatics analysis before IHC evaluation. In our bioinformatics analysis, we identified DOK3 as an important transcript in the immune response; higher expression levels of DOK3 were positively associated with immunosuppressive M2-like TAM in OSCC (*r* = 0.72, *P* < 0.001), which is in line with the findings of Liu et al. [35] in neurogliomas. In addition, the effect of DOK3 expression on M2-TAMs infiltration significantly increased after *P. gingivalis* treatment (*P* < 0.0001). These results were further validated by IHC analysis. Other markers for M2-TAM (*e.g.*, CD68^+^, CD163^+^, and CD204^+^) expressed in the local milieu of OSCC stromal spaces were confirmed by Petruzzi et al. [17]. Therefore a novel marker of CD206 was used to stain M2-TAMs in our immunohistochemical assessment [36]. We found that *P. gingivalis* immunoexpression levels were positively associated with DOK3 and CD206^+^ TAM immunoexpression levels, suggesting that *P. gingivalis* can affect onco-immunity in OSCC by increasing DOK3 and M2-TAM expression.

Bolz et al. [37] identified the bacterial spectra on the surface of OSCC in comparison to oral mucosa of patients with a higher risk to emerge an OSCC and a healthy control group; and they reported gram-negative anaerobes provided by biofilms on OSCC surfaces play a decisive role in the development of postoperative infections in patients with OSCC. However, currently no synthesized evidence from meta-analysis has demonstrated the prevalence rate of *P. gingivalis* and its association with OSCC prognosis. A few studies have reported that infection with *P. gingivalis* correlates with poor prognosis in patients with oral cancer [12, 29], one of which is the saliva sample type, and the densities of *P. gingivalis* are not stratified. In the present study, based on different staining intensities, we discovered that OSCC patients with strong immunoexpression levels of *P. gingivalis* had a worse prognostic outcome than those with the weak immunoexpression levels (HR = 1.674, 95%CI 1.216–4.142, *P* = 0.012), which enhances the existing conclusions. Similarly, detection with DOK3 (HR = 1.881, 95%CI 1.433–3.457, *P* = 0.042), and M2-TAM (HR = 1.649, 95%CI 0.824–3.082, *P* = 0.034) strong immunoexpression levels were associated with worse prognosis for OSCC patients.

Oral hygiene habits are being increasingly examined as explanatory factors for oral cancer. However, the relationship remains complex given the confounding effects of established determinants that are prevalent, as well as broader issues including a lack of access to positive oral health awareness and advanced healthcare facilities. Another key message from IHC analysis of DOK3 and M2-TAMs in *P. gingivalis*-infected OSCC immunomicroenvironment concluded that both strong staining of DOK3 and M2-TAMs exhibited significant associations with poor oral hygiene habits and severe dysphagia (Table 2). Likewise, a case–control study carried out by Gupta et al. [23] showed that reported habits of poor oral hygiene were significantly associated with an increased risk of oral cancer, after adjustment for other known risk factors. However, some medical co-morbidities – including hypertension and diabetes mellitus – may contribute to poor oral hygiene status [38]. In addition, whether a positive association between poor oral hygiene, tumor-infiltrated M2-TAMs and the risk of OSCC can be observed should be further investigated. Critically, tolerable oral diet without severe preoperative dysphagia is necessary to appraise oropharyngeal function, especially for the patients with floor of the mouth SCC [39]. The impact of early dysphagia should not be underestimated, even though preoperative dysphagia score exhibited no significant relation with the clinicopathological data and follow-up. By considering swallowing impairment at the primary therapy patients can profit concerning survival and comorbidity.

## Conclusion

In conclusion, for the first time, the relevance between different immunohistochemical intensities of *P. gingivalis* in OSCC tissue and clinicopathological characteristics and significance in prognosis was analyzed to better understand the participation of *P. gingivalis* in the OSCC immune microenvironment. Based on bioinformatics analyses, DOK3 was identified as the key DEG in the TME of OSCC infected with *P. gingivalis*, and its effect on TAM infiltration was significantly increased after *P. gingivalis* treatment. Furthermore, strong expression levels of DOK3 and M2-TAM were correlated with worse prognosis in patients with OSCC. Collectively, *P. gingivalis*, DOK3, and M2-type TAM could be considered as three novel independent risk factors for predicting the prognosis of OSCC. However, more basic researches on the molecular mechanism of the OSCC microenvironment in *P. gingivalis* infection need to be conducted in the future.

### Supplementary Information

**Supplementary Material 1. **

## Data Availability

Data is provided within the manuscript or supplementary information files.

## Abbreviations

AJCC: American Joint Committee on Cancer
CI: Confidence intervals
CSI: Cumulative survival rate
DEG: Differentially expressed gene
DOK3: Downstream of kinase 3
GEO: Gene Expression Omnibus
HR: Hazard ratios
OSCC: Oral squamous cell carcinoma
*P. **gingivalis*: *Porphyromonas* *gingivalis*
TAM: Tumor-associated macrophage
TCGA: The Cancer Genome Atlas
TME: Tumor microenvironment
UICC: Union for International Cancer Control
