# Supplementary material for: Association of DOK3 and infiltrated tumor-associated macrophages with risk for the prognosis of Porphyromonas gingivalis-infected oral cancer: a 12-year data analysis of 200 patients from a tertiary teaching hospital, Urumqi, China
Source: BMC Cancer. 2024 Apr 26;24:534. doi: 10.1186/s12885-024-12300-y (PMC11055382; doi:10.1186/s12885-024-12300-y)
Supplement: Supplementary file 1 — Supplementary Material 1. [file 12885_2024_12300_MOESM1_ESM.docx]

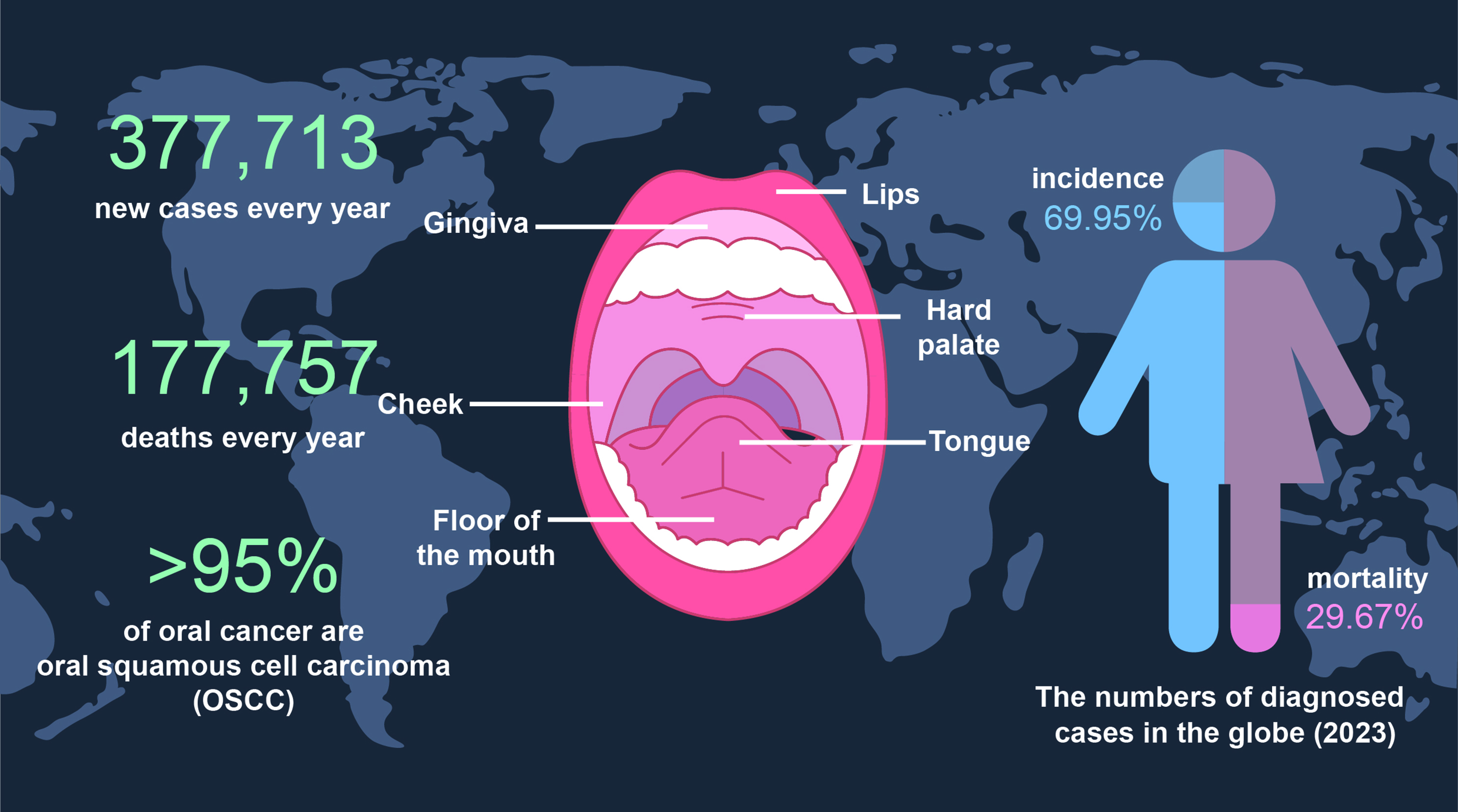


Supplementary Figure 1. The epidemiology of OSCC in 2023 globally (modified figure from <https://2020.igem.org/Team:CSMU_Taiwan/Description>).

Supplementary Table 1. Baselined information of all the included OSCC subjects (n = 200) according to different distributions.

| Clinical pathologic variables | No. of patients (%) |
| --- | --- |
| Sex |  |
| Male | 100 (50.0) |
| Female | 100 (50.0) |
| Age, years |  |
| < 60 | 73 (36.5) |
| ≥ 60 | 127 (63.5) |
| Survival status |  |
| Alive | 133 (66.5) |
| Dead | 67 (33.5) |
| Tobacco smoking |  |
| No | 68 (34.0) |
| Yes | 132 (66.0) |
| Alcohol consumption |  |
| No | 90 (45.0) |
| Yes | 110 (55.0) |
| Body mass index |  |
| < 22.5 | 132 (66.0) |
| ≥ 22.5 | 68 (34.0) |
| Baseline severe dysphagia  (Gr.3-6)^a^ |  |
| No | 129 (64.5) |
| Yes | 71 (35.5) |
| Diet |  |
| Vegetarian | 19 (9.5) |
| Non-vegetarian | 181 (90.5) |
| Milk & dairy products |  |
| Never | 2 (1.0) |
| Less than once a week | 29 (14.5) |
| More than once a week | 169 (84.5) |
| Clinical stage^b^ |  |
| I~II | 100 (50.0) |
| III~IV | 100 (50.0) |
| T stage^b^ |  |
| T1~T2 | 119 (59.5) |
| T3~T4 | 81 (40.5) |
| N stage^b^ |  |
| N0 | 135 (67.5) |
| N (+) | 65 (32.5) |
| Differentiation |  |
| Well | 129 (64.5) |
| Moderate | 49 (24.5) |
| Poor | 22 (11.0) |
| Recurrence |  |
| Yes | 31 (15.5) |
| No | 169 (84.5) |
| Oral hygiene habits^c^ |  |
| Good | 67 (33.5) |
| Average | 110 (55.0) |
| Bad | 23 (11.5) |
| Periodontal condition |  |
| Well | 56 (28.0) |
| Poor | 144 (72.0) |
| Neck dissection |  |
| No | 137 (68.5) |
| Yes | 63 (31.5) |
| Treatment |  |
| Surgery | 63 (31.5) |
| Radiotherapy | 59 (29.5) |
| Chemoradiation/comprehensive | 78 (39.0) |

**a**—Grade of dysphagia according to the standard symptom scale (doi: 10.4103/0973-1482.63563). In subjective dysphagia, evaluation score ranges from 0 to 6. Score 0 suggests no dysphagia and score 6 suggests ‘nothing by mouth’.

**b**—According to the 8th edition of the American Joint Committee on Cancer/ the International Union Against Cancer staging system.

**c**—The composite oral hygiene score (Gupta B et al., 2017), ranging from 0 to 6 (with a score of 4 or more indicating poor oral hygiene; 2 to 3, reasonable; 1 or less indicating good hygiene), aimed to capture oral hygiene habits and intra-oral examination findings for each study participant by summing up the following states: bleeding gums (no = 0, yes = 1); frequency of cleaning teeth (> 2 times a day = 0, ≤ once a day = 1); instrument used for cleaning (toothbrush = 0, finger or other = 1); substance used for cleaning (toothpaste/toothpowder = 0, other = 1); wearing dentures (no = 0, yes = 1); dental check-ups (rare = 0, only when in pain = 1); missing teeth (≤ 5 = 0, > 5 = 1).

Abbreviations: Gr, Grade

Supplementary Table 2. The intersection between GSE138206 and GSE24897 had 34 common differentially-expressed genes, including four downregulated genes and 30 upregulated genes.

(A) GSE138206

|  | GSM4101931 | GSM4101932 | GSM4101933 | GSM4101934 | GSM4101935 | GSM4101936 | GSM4101925 | GSM4101926 | GSM4101927 | GSM4101928 | GSM4101929 | GSM4101930 |
| --- | --- | --- | --- | --- | --- | --- | --- | --- | --- | --- | --- | --- |
| CXCL3 | 5.496417 | 5.032738 | 5.872254 | 5.297815 | 5.402901 | 5.415401 | 6.92492 | 6.258448 | 8.673856 | 7.244863 | 5.964746 | 6.76974 |
| CHI3L1 | 8.104742 | 7.601237 | 7.475626 | 5.521954 | 7.782127 | 7.915112 | 9.450806 | 9.612672 | 12.71203 | 8.556811 | 11.99954 | 10.93292 |
| CXCL10 | 6.493649 | 7.301368 | 7.61318 | 7.75569 | 8.760006 | 8.243999 | 10.06861 | 10.11514 | 11.05302 | 10.42005 | 9.771999 | 11.89843 |
| TNFAIP6 | 8.698044 | 6.839776 | 8.977162 | 7.658662 | 7.567771 | 7.390776 | 9.640035 | 7.817792 | 11.38498 | 9.308188 | 8.689651 | 9.865454 |
| MSC | 5.539567 | 5.428245 | 5.719348 | 5.950282 | 5.492626 | 5.8582 | 7.165112 | 6.277469 | 7.625364 | 7.301049 | 6.580212 | 7.149032 |
| CCL4 | 6.853447 | 7.063481 | 9.439912 | 7.915845 | 7.307958 | 8.613307 | 7.785293 | 8.825118 | 11.33417 | 9.164966 | 9.067317 | 10.1586 |
| SLAMF1 | 4.612028 | 4.618654 | 4.587311 | 5.345708 | 4.218715 | 6.441359 | 5.834553 | 5.618008 | 7.063481 | 5.933504 | 5.571924 | 6.719349 |
| MIR155 | 4.763122 | 4.892413 | 5.188303 | 5.162231 | 4.042098 | 6.863504 | 6.297899 | 6.33904 | 7.22968 | 6.367042 | 5.812055 | 7.751898 |
| GPR84 | 4.79869 | 4.716946 | 5.45341 | 4.494825 | 4.474143 | 4.987251 | 4.673974 | 5.676177 | 8.925805 | 6.453225 | 6.103463 | 6.444184 |
| PARP9 | 8.216142 | 7.76469 | 8.535474 | 8.486523 | 7.340353 | 8.299907 | 9.330336 | 9.416209 | 9.161553 | 10.40954 | 9.021845 | 9.742422 |
| CXCL9 | 6.966746 | 6.33904 | 7.216043 | 8.54652 | 7.290868 | 7.715593 | 10.65779 | 10.20435 | 9.852456 | 10.03058 | 9.409544 | 11.75127 |
| IRF8 | 7.095005 | 6.723802 | 8.12249 | 8.304835 | 6.774953 | 9.246147 | 8.534294 | 7.82656 | 9.31654 | 9.277322 | 8.310601 | 9.797145 |
| APOBEC3G | 7.423801 | 7.630035 | 7.969256 | 8.435093 | 6.733695 | 8.987363 | 9.313202 | 8.319228 | 9.200819 | 9.027593 | 7.912517 | 10.41341 |
| DRAM1 | 8.08342 | 7.721993 | 8.72012 | 7.681063 | 7.715084 | 8.040563 | 9.031072 | 8.629647 | 10.15963 | 8.6658 | 9.128098 | 9.681584 |
| TLR2 | 7.563518 | 6.95322 | 7.43233 | 6.676333 | 7.673294 | 7.585961 | 8.515773 | 7.520905 | 9.908756 | 8.32949 | 9.911473 | 8.339602 |
| GBP1 | 8.077585 | 8.211418 | 9.022895 | 9.771999 | 9.970803 | 8.778828 | 9.91893 | 9.709625 | 10.19197 | 9.882177 | 9.535517 | 11.25556 |
| IL7R | 7.599234 | 7.484827 | 7.439824 | 8.554728 | 7.518107 | 8.956625 | 9.65709 | 9.055024 | 9.259029 | 9.834925 | 9.313202 | 9.954127 |
| CCL18 | 8.038061 | 5.609915 | 8.879631 | 6.974618 | 7.83128 | 9.264103 | 10.89029 | 10.92686 | 12.12075 | 9.572495 | 11.91991 | 12.21363 |
| HMGCS1 | 7.0509 | 6.215751 | 8.9984 | 8.495735 | 6.123126 | 8.514994 | 9.091063 | 7.289779 | 9.627379 | 7.420456 | 9.327281 | 9.017736 |
| KLF7 | 7.766127 | 7.109449 | 7.667793 | 7.475288 | 7.780407 | 7.019222 | 9.078505 | 9.218745 | 8.137703 | 8.233711 | 8.670816 | 8.215165 |
| CHST2 | 6.220132 | 6.68576 | 7.071073 | 7.014832 | 6.897964 | 7.614189 | 9.017736 | 9.968158 | 8.878001 | 8.46891 | 8.410752 | 9.164966 |
| ANKLE2 | 7.261125 | 6.518178 | 6.632529 | 6.70484 | 7.329471 | 7.455497 | 8.332035 | 8.155316 | 7.275081 | 7.380769 | 8.364007 | 8.458232 |
| TPBG | 9.138645 | 9.140504 | 9.719538 | 8.5298 | 9.119317 | 8.803816 | 10.58502 | 11.16613 | 10.0605 | 10.22916 | 10.43206 | 9.714081 |
| FCGR2A | 7.409984 | 6.868998 | 8.330785 | 8.076138 | 7.140408 | 7.752623 | 8.216142 | 8.479742 | 10.24557 | 8.914169 | 10.19197 | 9.515082 |
| ABCA1 | 8.421566 | 7.92585 | 7.91463 | 8.429383 | 8.058429 | 7.471003 | 9.518861 | 9.501431 | 9.637712 | 9.094299 | 9.236789 | 9.445846 |
| LRP12 | 6.755665 | 6.825052 | 6.916577 | 6.462934 | 7.090177 | 6.021981 | 8.114699 | 8.305922 | 7.737891 | 7.944254 | 7.820768 | 7.403046 |
| PARP14 | 7.460342 | 7.191297 | 7.509993 | 7.859469 | 7.119076 | 7.910688 | 9.541881 | 9.0475 | 8.99068 | 9.82147 | 8.709365 | 9.509099 |
| HSD11B1 | 5.882841 | 5.842774 | 5.273009 | 5.547358 | 6.180686 | 5.585691 | 6.292905 | 6.826289 | 7.088781 | 6.30672 | 7.123597 | 6.857426 |
| RNF213 | 7.979839 | 7.443649 | 7.968907 | 8.091991 | 7.53343 | 7.79484 | 9.369931 | 8.245757 | 8.869448 | 9.784581 | 8.402792 | 9.061245 |
| DOK3 | 6.221845 | 6.481914 | 7.428404 | 7.030157 | 5.902442 | 8.144004 | 7.042634 | 7.360318 | 9.505179 | 7.590112 | 7.843095 | 8.316782 |
| OBFC1 | 9.785902 | 9.890232 | 9.940647 | 10.21716 | 9.182292 | 9.014363 | 7.807488 | 7.998459 | 8.398474 | 8.258674 | 8.78993 | 8.086325 |
| CHMP4C | 10.14234 | 10.52927 | 9.828366 | 9.674555 | 10.2919 | 8.725453 | 7.952568 | 9.009634 | 8.012737 | 7.504534 | 8.693966 | 8.205487 |
| AHNAK | 9.957226 | 9.961946 | 9.436826 | 10.56963 | 9.444005 | 8.89754 | 8.133387 | 9.223758 | 7.604353 | 9.034283 | 9.407983 | 8.008511 |
| TACSTD2 | 7.300731 | 6.381969 | 7.400718 | 7.393933 | 6.428954 | 6.379054 | 4.658961 | 5.604814 | 5.184015 | 6.213465 | 5.716324 | 5.181628 |

(b) GSE24897

|  | GSM612262 | GSM612263 | GSM612264 | GSM612265 | GSM612266 | GSM612267 |
| --- | --- | --- | --- | --- | --- | --- |
| CXCL3 | 6.2951695 | 6.1727005 | 7.135647833 | 12.759824 | 12.74826383 | 12.44123917 |
| CHI3L1 | 8.622451333 | 8.756189167 | 9.599034 | 14.1178045 | 14.06723433 | 14.3968535 |
| CXCL10 | 8.9818455 | 7.6878685 | 7.939872667 | 11.7852135 | 11.97731417 | 10.98286933 |
| TNFAIP6 | 10.8726295 | 11.04627217 | 8.8561235 | 13.09984317 | 13.35782033 | 13.271751 |
| MSC | 7.6848355 | 7.571303833 | 9.1609375 | 10.37501533 | 10.25014833 | 10.41522317 |
| CCL4 | 12.2795175 | 12.04879117 | 11.82723567 | 14.22185333 | 14.348787 | 14.18826383 |
| SLAMF1 | 7.8576185 | 8.054215667 | 5.156125 | 9.494578833 | 9.376351833 | 8.725226333 |
| MIR155 | 9.8206275 | 9.492887667 | 8.600243667 | 11.2532735 | 11.41249967 | 11.21234933 |
| GPR84 | 7.752288167 | 7.3696475 | 7.840137167 | 9.729037833 | 9.804053 | 9.284496167 |
| PARP9 | 6.893763833 | 6.961245667 | 8.1822755 | 9.306086167 | 9.193955833 | 9.098857167 |
| CXCL9 | 8.698348833 | 8.055299167 | 6.964276667 | 9.710879667 | 9.957512 | 9.4881035 |
| IRF8 | 9.8848645 | 9.786097333 | 9.132057667 | 11.50972917 | 11.5162715 | 11.14448833 |
| APOBEC3G | 6.865411333 | 7.1623495 | 7.929764833 | 10.06554883 | 7.867463667 | 9.019538167 |
| DRAM1 | 10.164778 | 10.29882033 | 9.712619 | 11.897555 | 11.73558767 | 11.233512 |
| TLR2 | 9.641708833 | 9.427467833 | 9.318273333 | 11.24831617 | 11.12984133 | 10.6737175 |
| GBP1 | 5.676146167 | 5.365842667 | 3.786382667 | 6.544010167 | 6.885682167 | 5.792974667 |
| IL7R | 8.222447833 | 8.9256705 | 7.563649667 | 9.5629455 | 9.863299667 | 9.639032667 |
| CCL18 | 8.289376167 | 8.094368333 | 6.803397167 | 9.246273 | 9.451410167 | 8.688368833 |
| HMGCS1 | 7.312819667 | 6.9166375 | 6.8306455 | 7.961821333 | 7.959674167 | 9.269934167 |
| KLF7 | 6.9420985 | 7.244558667 | 7.002024333 | 8.165227833 | 8.413713167 | 8.6876475 |
| CHST2 | 7.430583167 | 7.737022333 | 7.960659 | 8.790235333 | 8.646910833 | 9.714487333 |
| ANKLE2 | 7.4654295 | 7.430277 | 7.60297 | 8.865343167 | 8.568197667 | 8.953177333 |
| TPBG | 7.144455833 | 6.949664333 | 6.754444667 | 7.925107167 | 8.013109833 | 8.766021 |
| FCGR2A | 7.758789333 | 8.029947833 | 6.431426 | 8.785337333 | 8.84096325 | 8.397434167 |
| ABCA1 | 8.221916 | 7.979658167 | 8.478627 | 9.594603167 | 9.522618333 | 9.363109667 |
| LRP12 | 8.396585333 | 7.896726333 | 9.094221333 | 9.691019667 | 9.725980667 | 9.650783833 |
| PARP14 | 8.830575833 | 8.950142333 | 8.0223955 | 9.931036667 | 9.7716055 | 9.736020667 |
| HSD11B1 | 8.043738833 | 8.198378167 | 7.201668333 | 8.834908333 | 9.099357333 | 8.990558167 |
| RNF213 | 8.462891667 | 8.425387167 | 7.671043167 | 9.6919215 | 9.357156333 | 8.9678935 |
| DOK3 | 6.747575 | 6.555355 | 6.448478 | 8.223771 | 8.144975 | 8.941982 |
| OBFC1 | 7.449995833 | 7.616885667 | 7.021763 | 6.303715667 | 6.077027333 | 6.448478167 |
| CHMP4C | 7.213187833 | 6.997130167 | 5.881211333 | 5.4893575 | 5.324637167 | 5.691561417 |
| AHNAK | 8.059514333 | 8.396585333 | 9.968622167 | 6.8810455 | 6.468423167 | 7.396791333 |
| TACSTD2 | 9.127684833 | 9.464861333 | 8.703828333 | 6.722602333 | 6.5970125 | 6.870000833 |
